# Coupling BCI and cortical stimulation for brain-state-dependent stimulation: methods for spectral estimation in the presence of stimulation after-effects

**DOI:** 10.3389/fncir.2012.00087

**Published:** 2012-11-16

**Authors:** Armin Walter, Ander R. Murguialday, Wolfgang Rosenstiel, Niels Birbaumer, Martin Bogdan

**Affiliations:** ^1^Department of Computer Engineering, Wilhelm-Schickard-Institute, Eberhard Karls Universität TübingenTübingen, Germany; ^2^Institute of Medical Psychology and Behavioural Neurobiology, University Hospital TübingenTübingen, Germany; ^3^Health Technologies Department, TECNALIASan Sebastian, Spain; ^4^Ospedale San Camillo, IRCCSVenice, Italy; ^5^Department of Computer Engineering, University of LeipzigLeipzig, Germany

**Keywords:** brain-computer interfaces, cortical stimulation, spectral estimation, brain-state-dependent stimulation, autoregressive models

## Abstract

Brain-state-dependent stimulation (BSDS) combines brain-computer interfaces (BCIs) and cortical stimulation into one paradigm that allows the online decoding for example of movement intention from brain signals while simultaneously applying stimulation. If the BCI decoding is performed by spectral features, stimulation after-effects such as artefacts and evoked activity present a challenge for a successful implementation of BSDS because they can impair the detection of targeted brain states. Therefore, efficient and robust methods are needed to minimize the influence of the stimulation-induced effects on spectral estimation without violating the real-time constraints of the BCI. In this work, we compared four methods for spectral estimation with autoregressive (AR) models in the presence of pulsed cortical stimulation. Using combined EEG-TMS (electroencephalography-transcranial magnetic stimulation) as well as combined electrocorticography (ECoG) and epidural electrical stimulation, three patients performed a motor task using a sensorimotor-rhythm BCI. Three stimulation paradigms were varied between sessions: (1) no stimulation, (2) single stimulation pulses applied independently (open-loop), or (3) coupled to the BCI output (closed-loop) such that stimulation was given only while an intention to move was detected using neural data. We found that removing the stimulation after-effects by linear interpolation can introduce a bias in the estimation of the spectral power of the sensorimotor rhythm, leading to an overestimation of decoding performance in the closed-loop setting. We propose the use of the Burg algorithm for segmented data to deal with stimulation after-effects. This work shows that the combination of BCIs controlled with spectral features and cortical stimulation in a closed-loop fashion is possible when the influence of stimulation after-effects on spectral estimation is minimized.

## 1. Introduction

Cortical stimulation is being used to study cortical function, e.g., (Matsumoto et al., 2007). In clinical settings, it is employed for surgical planning (Lefaucheur and de Andrade, 2009) and therapy (Tsubokawa et al., 1991). Furthermore, preliminary studies on the use of cortical stimulation for stroke rehabilitation which used stimulation together with physiotherapy in order to modulate cortical excitability have been conducted (Brown et al., 2008; Levy et al., 2008). Taking the current brain activity of the patient into account when selecting stimulation parameters has been proposed as a possible improvement (Plow et al., 2009). Such an activity-dependent stimulation paradigm has been used by Jackson et al. (2006), who were able to show that cortical microstimulation associated in time with brain activity during a motor task can induce neural reorganization lasting for several days after stimulation in primates.

The effects of transcranial magnetic stimulation (TMS) as well depend on brain states of the stimulated person (Mitchell et al., 2007). Recently, Bergmann et al. (2012) applied TMS coupled to electroencephalography (EEG) to investigate the dependency of stimulation effects on the phase of slow EEG oscillations during sleep. In general, such activity-dependent or brain-state-dependent stimulation (BSDS) paradigms allow to investigate cortical networks at specific activation levels, making BSDS a potentially useful tool in cognitive neuroscience (Jensen et al., 2011) as well as in clinical studies improving consistency of the stimulation effects (Plow et al., 2009).

For effective BSDS, reliable decoding of the brain-state from the ongoing brain activity is necessary. Over the last decades in the field of brain-computer interfaces (BCIs) several different strategies were investigated (Birbaumer et al., 1999; Birbaumer and Cohen, 2007). Especially in the case of movement-related brain states during active or imagined limb movements, spectral power has been shown to be useful for their decoding. In particular, event-related (de-) synchronization of sensorimotor rhythms is an informative measure for discriminating movement and resting states (Wolpaw et al., 2002). Therefore, if one wants to combine BSDS with a movement task, one has to minimize the interference of the stimulation on the estimation of the spectral features to detect the brain-state properly.

**Figure 1:**
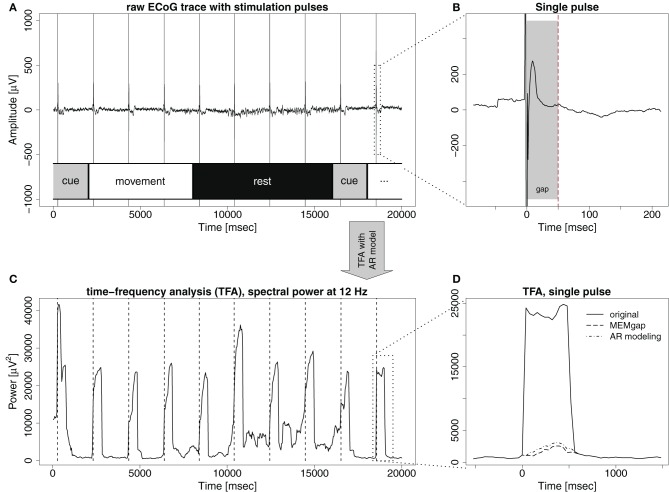
**The effect of stimulation pulses on time-frequency analysis (TFA) with AR models**. **(A)** Example trace of ECoG data with intermittent stimulation pulses. Each pulse is visible as a sharp, strong artefact in the signal. The lower part of the illustrates the phases of the trial over the course of the recording: *cue*: an auditory cue, *movement*: patient attempts to move the hand, and *rest*: patient relaxes. **(B)** A zoom on the last stimulation pulse visible in **(A)**, also displaying an evoked potential, peaking 13 ms after the pulse. If the gray-shaded area up to the dashed line is defined as a gap, both the stimulation artefact and the strongest evoked components are covered. **(C)** Time-course of the power at 12 Hz of the signal displayed in **(A)**, resulting from a time-frequency analysis with auto-regressive models (order 16) when a window of 500 ms is shifted in 40 ms steps over the data. Hence, a single stimulation pulse distorts the spectrum for the next 500 ms because it remains in the data window. **(D)** Zoom on the region of the last stimulation pulse. Power at 12 Hz without stimulus processing (solid line) and when the gap is defined as in **(B)** and either MEMgap (dashed line) or AR modeling with order 16 (dashed-and-dotted line) are applied to deal with it.

The stimulation effects involve problems with spectral estimation caused by the stimulation artefact and the evoked neural activity. A stimulation pulse evokes an artefact in the signal (Figure 1A) with an amplitude in the range of several hundred millivolts or even volts, thus often exceeding the dynamic range of the amplifier (Veniero et al., 2009). In the vicinity of stimulation, evoked potentials are recorded (Figure 1B) which can reach amplitudes of several hundred microvolts (Matsumoto et al., 2007). Thus, if an analyzed window contains a stimulation pulse, the estimation of the spectrum of this window is difficult, because it is not stationary. This is evident in Figure 1C, showing that each stimulation pulse results in strong jumps in the estimated spectral power. Waiting long enough after the pulse is one solution. This approach results in non-continuous brain-state decoding with waiting periods after a stimulus of at least several hundred milliseconds. It dictates a longer inter-stimulus interval (ISI), because a robust estimate of the brain-state is needed before the next pulse can be applied.

If small ISIs and/or continuous decoding of the brain-state is necessary, methods that enable spectral estimation of data containing stimulation after-effects are mandatory. One potential solution for this, which has been used mainly in offline studies (no BSDS), is to separate the stimulation effects from the signal, as for example in Litvak et al. (2007). This places restrictions on the recording setup, such as the need for an amplifier with high dynamic range to cover the entire amplitude of the artefact and it is unclear whether such a procedure can be performed online without resulting in residual artefacts which would still lead to distortions of the spectrum. We present in this paper another solution suitable for online BSDS: we ignore the short segment of data dominated by the after-effects of stimulation when estimating the spectrum, leaving us with the challenge to estimate the spectrum when portions of the data are missing from a continuous data flow. We term such an excluded data segment a *gap*. In online experiments, using either signals synchronized with the stimulator or a peak detection algorithm, one can mark a sample before the stimulation pulse as the beginning of the gap. The number of following samples marked as belonging to the gap (i.e., the *gap size*) should be chosen in advance such that the gap, ideally, encloses just the stimulation artefact, and the largest evoked components. The dashed line and the dashed-and-dotted line in Figure 1D show the results of two approaches introduced in this work to extract the spectral power when the artefacts are masked by the gap shown in Figure 1B. They are much closer to the power before and after the stimulus, compared to the power without any processing of the stimulus (solid line).

In this paper we compare different online brain-state decoding methods on their suitability to perform spectral estimation using autoregressive (AR) models on data containing stimulation pulses and gaps. We consider here only stimulation paradigms with pulsed stimuli and restrict ourselves to data acquired with EEG or electrocorticography (ECoG) and stimulation performed using TMS or epidural electrodes. First, we introduce the methods for spectral estimation in the presence of gaps and investigate the effects of parameter estimates such as AR model order and gap size on the resulting spectrum. We present results from a simulation study in which gaps are artificially inserted into a BCI data set recorded without stimulation. We then show the different results of the algorithms on short data segments of two BCI training experiments, one with simultaneous TMS and one with simultaneous epidural electrical stimulation to illustrate the effects of cortical stimulation on spectral estimation and the results of correcting stimulation after-effects. Finally, we investigate the separability of intended hand movement and rest for different experimental paradigms (no stimulation, open-loop, or closed-loop stimulation) using non-invasive and invasive data during BCI experiments in three chronic stroke patients.

## 2. Methods

### 2.1. Participants

**Figure 2:**
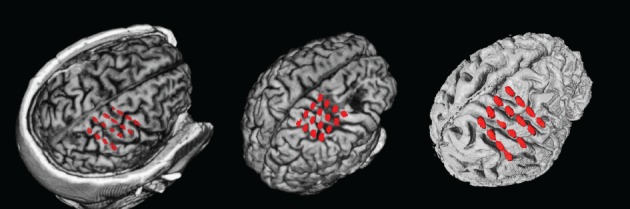
**ECoG electrode positions from overlay of MRI and post-surgical CT for the three patients**. From left to right: P1–P3.

Data was recorded from three chronic stroke patients (Table 1) suffering from paresis of the left hand. None of the patients was able to produce voluntary finger movements with the left hand.All procedures were approved by the local ethics committee of the medical faculty of the university hospital in Tübingen. Each stroke patient was implanted with 16 epidural platinum iridium disk electrodes (Resume II, Medtronic, Fridley, USA) with a contact diameter of 4 mm placed over the ipsilesional S1, M1, and pre-motor cortex on four strips with an inter-electrode center-to-center distance of 10 mm. They were arranged in a 4 × 4 grid-like pattern (Figure 2). During pre-surgical evaluation, all subjects completed the task described below with combined EEG-TMS (*non-invasive case*) and repeated the same task after the surgery using electrical epidural stimulation and recordings from the implanted electrodes (*invasive case*). The BCI and stimulation experiments were conducted during a period of 4 weeks following the implantation.

**Table 1:** **Patient characteristics**.

**Patient**	**Age (y)**	**Sex**	**Months lapsed since injury**	**Paralysis**	**Infarct side**	**Lesion**	**Affected area**
P1	56	M	80	Left	Right subcortical and cortical	Basal ganglia hemorrhage	Putamen, internal capsule, insula, opercular part of inferior frontal gyrus
P2	52	M	159	Left	Right subcortical and cortical	MCA territory infarct (frontal)	Frontal lobe including motor cortex (M1), parietal lobe including somatosensory cortex (S1)
P3	63	F	71	Left	Right subcortical and cortical	Basal ganglia hemorrhage	Head of striate body, lentiform nucleus, thalamus, whole internal capsule, insula, frontal lobe

### 2.2. Task

The patient was facing a 19″ monitor. The left upper limb of the patient was fixed using two straps, one at the forearm and one around the wrist and magnets fixed the fingertips to the actuators of a mechatronic hand orthosis (Tyromotion Amadeo HTS, Graz, Austria). This device was controlled by a BCI and moved the fingers of the paralyzed hand between an opened and a closed position. The range of the movement was adjusted in each session (Ramos-Murguialday et al., 2012) because it was limited by the spasticity of the patient. Each trial of the task consisted of three phases: preparation (2 s), feedback (6 s), and rest (8 s). During preparation, the subject received an auditory cue (“Left Hand”) but was instructed to wait with the execution until the next auditory command (“Go!”) was given at the start of the feedback phase. During the feedback phase starting with a closed position of the left hand, the patient had to try to open the left hand until the end of the feedback phase. At that point, another auditory cue (“Relax!”) was given. During the rest period, the left hand of the patient was returned to its original closed position (2–3 s) and the patient was instructed to relax. An experimental session was divided into a 4–16 runs, each of these consisting of 11 trials. Runs with clear non-stimulation-related artefacts (e.g., amplifier saturation) on the analyzed channels were excluded from further analysis, resulting in a minimum of three runs per session for analysis and an average of 8.7 ± 4.3.

### 2.3. Electrophysiological recording

Both EEG and ECoG were recorded with monopolar 32-channel amplifiers (BrainAmp MR plus, BrainProducts, Munich, Germany) with a sampling rate of 1000 Hz. The data was acquired in a packet-wise fashion, where the recording computer received every 40 ms one packet of data consisting of 40 samples per channel. The same behavior was modeled in our simulations of an online BCI. A high-pass filter with a cutoff frequency at 0.16 Hz and a low-pass filter with a cutoff frequency at 1000 Hz were applied. We recorded 32 channels of EEG in the standard 10–10 system, referenced to FCz, using circular Ag-AgCl electrodes. ECoG data was referenced to an electrode at the medio-frontal corner of the electrode grid over pre-motor cortex. Signal acquisition, signal processing and control of the orthosis and (if present in the experiment) the TMS or electrical stimulator were performed using the general-purpose BCI framework BCI2000 (http://www.bci2000.org) (Schalk et al., 2004) extended with custom-developed features for the control of these devices.

### 2.4. Stimulation

We applied stimulation in the non-invasive case over the hotspot for *extensor digitorum communis* (EDC) activity, identified by a standard mapping paradigm (Wassermann et al., 2008). TMS was applied with a figure-of-eight coil (NeXstim, Helsinki, Finland) with single biphasic pulses (sinusoidal coil current, positive phase first, pulse width 280 μs) and an intensity of 110% of the resting motor threshold. The ISI of successive pulses was set to 3 s.

For epidural electrical stimulation we used single biphasic anodal square-wave pulses with a length of 500 μs. Stimulation intensity was selected individually per patient and session and chosen to reliably evoke MEPs on the paretic upper limb of the patient. The minimum ISI was set to 2 s in most experiments except when stimulation was applied coupled with the BCI output. In this case, a minimum ISI of 500 ms was chosen. The pulses were applied using a constant current stimulator (STG4008, Multichannel systems, Reutlingen, Germany) with the anode as the epidural electrode that evoked the strongest MEPs on the left upper limb and the cathode being a 50 × 90 mm adhesive electrode placed on the left clavicle of the patient. The current source of the stimulator was switched off 2 s after the last stimulation pulse if no other pulse was triggered before due to a software error, leading to a small but visible step in the recorded signal (Figure 1B).

### 2.5. Autoregressive (AR) models

A popular choice for spectral estimation in BCI research is to use an AR model for which the coefficients are estimated with the maximum entropy method (Krusienski et al., 2006; McFarland and Wolpaw, 2008). An AR model can be viewed as a linear predictor of the signal samples *x*(*t*_*k*_), defined as:

$$x(t_{k})=\sum_{i\;=\;1}^{p}c_{i}x(t_{k\;-\;i})+e$$

where *p* is the order of the model and *e* a sample of a white noise process. If one uses a continuous window of length *N* with *N* » *p* consisting of samples *x*(*t*_0_) to *x*(*t*_*N* − 1_), one could solve the following equations with a least-squares procedure to get the coefficients *c*_*i*_:

(1) $$\begin{array}{c} x(t_{k})=\sum_{i\;=\;1}^{p}c_{i}x(t_{k\;-\;i})\\ x(t_{k-p})=\sum_{i\;=\;1}^{p}c_{i}x(t_{k\;-\;p\;+\;i})\text{ for all }k=p,\ldots ,N-1 \end{array}$$

However, the resulting coefficients do not guarantee a stable AR model (de Waele and Broersen, 2000). Burg proposed a recursive algorithm for the solution of this system that provides stable models with less variance compared to least squares solutions and the Yule-Walker algorithm (Kay and Marple, 1981; de Waele and Broersen, 2000). The Burg algorithm computes the AR coefficients in *p* steps by evaluating in the *i*-th step the residuals of forward and backward prediction of the samples using the coefficients obtained in the (*i* − 1)-th step. It is described in Appendix 1.1 in more detail. Spectral estimation with AR models is briefly introduced in Appendix 1.2.

The Burg algorithm requires that the input data is sampled continuously without gaps, a condition which is shared by most of the other algorithms for AR model estimation. Therefore, we need to either fill or remove the gaps before applying one of these algorithms to our data or modify the AR model estimation algorithms to be usable for data with gaps.

### 2.6. Spectral estimation in the presence of gaps

This section contains a short description of the different algorithms we compare in this paper that deal with the pre-processing of data containing gaps for spectral estimation with AR models. The input for these algorithms are a segment of data and a vector that contains for each sample in the segment either a 1 (sample belongs to a gap, it has to be excluded from spectral estimation) or a 0 (sample is “clean”).

Four methods for dealing with gaps in the data are described below: (1) *linear interpolation*, (2) *AR modeling* which fill the gap with generated data, (3) the *joining of data segments* that removes the gap, and (4) a *modified Burg algorithm for segmented data*. After application of the methods (1)–(3), the standard Burg algorithm is used to estimate the AR model and the spectrum.

#### 2.6.1. Linear interpolation

We can bridge gaps in the data by linear interpolation between the last sample before and the first sample after the gap:

(2) $$\hat{x}(t_{g\;+\;k})=x(t_{g\;-\;1})+\frac{k+1}{l+1}\cdot \left(x(t_{g\;+\;l})-x(t_{g\;-\;1})\right),0\leq k\leq l-1$$

where *x* are the signal samples recorded at times *t*_*i*_, *l* is the length of the gap in samples and *t*_*g* − 1_ is the index of the last sample before the gap.

While this might work for offline analysis of a data set, in the case of online analysis during a BCI experiment, in which data is received in a sample- or packet-wise system, one might have not yet received the first clean sample after the gap when trying to produce an estimate for *x*(*t*_*g* + *k*_) within the gap. We used a simple approach to solve this problem which consists of filling the gap with the value of the last sample before the gap $\left(\hat{x}(t_{g\;+\;k})=x(t_{g\;-\;1})\right)$ as long as we have not received the packet containing the end of the gap and using linear interpolation for the rest of the gap otherwise. We term this approach *on-line compatible linear interpolation*.

#### 2.6.2. AR modeling

As a somewhat more sophisticated technique compared to linear interpolation, we generated data from an AR model to fill the gap. For this we used the coefficients *c*_*i*_ of the AR model estimated for the data window directly before the gap to predict the missing samples $\hat{x}$:

(3) $$\begin{array}{c} \hat{x}(t_{g\;+\;k})=\sum_{i\;=\;1}^{p}c_{i}x^{\prime }(t_{g+k-i})+\sigma \cdot e(t_{g+k}),\;0\leq k\leq l-1,\\ x^{\prime }(t_{g+j})=\{\begin{array}{ll} x(t_{g+j}) & \text{if }j<0\\ \hat{x}(t_{g+j}) & \text{otherwise} \end{array} \end{array}$$

*x*′ can refer to either actually recorded samples before the gap or estimated samples by the AR procedure. σ is the standard deviation of the white noise component in the estimated AR model and *e*(t) one value of a white noise process. While this approach has the property to generate data for the gap consistent with the previously measured data, one might prefer to use a mixture of AR modeling and linear interpolation for the online case. This would avoid jumps in the data when merging generated data within the gap with new samples acquired after the gap. These jumps occur for all AR model orders we have tested in our simulations (see Appendix 1.4 for details). We have used this combination here by performing AR extrapolation when information about the first sample after the gap was not available and using linear interpolation otherwise. The signal was received in packets with a length of 40 ms and for each packet, one of three actions were taken: (1) if a packet contained the start and the end of a gap, then linear interpolation was used to fill the gap. (2) If it contained only the start or if the whole packet was part of the gap, then the AR model was used as a linear predictor to fill the gap. (3) If it contained only the end of the gap, then the last sample of the last packet and the first sample after the gap were connected by linear interpolation.

#### 2.6.3. Joining two segments

If one chooses to ignore the information of the gap altogether when estimating the model, one might consider simply joining the two segments around the gap, therefore sacrificing information about the timing in the vicinity of the gap. In practice, this means that we update the data window only with those samples from a newly acquired data packet that do not belong to a gap. In order to keep the window size for spectral estimation constant, this has the consequence that older samples are used to compute the spectrum with this method compared to the other algorithms.

#### 2.6.4. Burg algorithm for segments (MEMgap)

For standard algorithms that compute the AR coefficients, the samples within the data window need to be continuous. We can make the least-squares estimation of the AR coefficients compatible with data containing gaps by eliminating all equations from (Equation 1) that contain samples from within a gap and then solving the rest of the equations for the coefficients *c*_*i*_. As the Burg algorithm (see Appendix 1.1) yields more stable AR models than the least-squares estimation, we modified it to work with gaps based on the Burg algorithm for segmented data proposed in de Waele and Broersen (2000). This was achieved by limiting the computation of forward and backward prediction errors in each step of the algorithm to those samples that are far enough away from a gap. In the remainder of this paper, this algorithm is called MEMgap (Maximum Entropy Method for data with gaps) for brevity. A detailed description of the algorithm is given in Appendix 1.3.

### 2.7. Simulations on clean data

To study empirically the influence of gaps on the estimated spectrum, we performed simulations on 12 data sets that were recorded without stimulation by artificially inserting gaps, then applying the methods described above to estimate the spectrum. The results of the different methods were compared with a reference time-frequency analysis obtained when using the original data set without gaps. Each data set has a length of 182 s. These data sets, each containing 11 trials, were recorded with ECoG in patient P1 in one experimental session. For clarity reasons, we restrict ourselves to one channel (an electrode over right M1). For spectral computation we kept the length of the window constant at 500 ms and the update rate at 25 Hz = 40 samples. We estimated the power at frequencies between 5 and 99 Hz in 2 Hz increments and varied for each method the gap size (0–100 ms in steps of 5 ms) and the model order (values: 16, 32, and 64). We computed the normalized bias, root mean squared error (RMSE) and variance (var) of the stimulus processing algorithms as follows:

$$\begin{array}{l} \text{ bias}(f)=\frac{\frac{1}{n}\sum_{i}\left(P\left(f,\;i\right)-P_{0}\left(f,\;i\right)\right)}{\bar{P_{0}(f)}}\\ \text{RMSE}(f)=\frac{\sqrt{\frac{1}{n}{\sum_{i}\left(P\left(f,\;i\right)-P_{0}\left(f,\;i\right)\right)}^{2}}}{\bar{P_{0}(f)}}\\ \text{ var}(f)=\text{Var}\left(\frac{P(f)-P_{0}(f)}{\bar{P_{0}(f)}}\right) \end{array}$$

*P*(*f*, *i*) is the spectral power of data window *i* for frequency bin *f*, *P*_0_(*f*, *i*) is the power of the original data window without gaps and $\bar{P_{0}(f)}$ is the average power of the full original recording without gaps for frequency bin *f*. *n* is the number of data windows that are affected by gaps (i.e., data windows where *P*(*f*, *i*) − *P*_0_(*f*, *i*) is not zero). *var*(*f*) is the variance of the difference between the power values of the original data and the power values of the data with gaps for all data windows affected by gaps and frequency bin *f*, divided by the average power for frequency bin *f* in the data set without gaps. For example, a normalized bias of −0.1 means that the estimated power after application of the stimulus processing algorithm is on average 10 % smaller than the power of the original data set if a gap is present.

The statistical evaluation of the spectral bias results in the simulations was performed as follows: we obtained the bias for each data set, resulting in 12 values, and performed a non-parametric Wilcoxon signed-rank test for zero median. If the *p*-value for this test was below 0.01, we regarded the bias as significant.

## 3. Results

First we show results of simulated gaps on data without stimulation to assess the influence of gaps and the stimulation-processing algorithms on the estimated spectrum. Then we illustrate the influence of real single TMS and epidural stimulation pulses on the spectrum if they are left untreated and how the methods of this paper deal with their after-effects. Finally, we apply the algorithms to data sets of BCI experiments with open-loop or closed-loop stimulation and investigate the effect of each method on the discrimination between the brain states during intended movements and rest.

### 3.1. Gap size

**Figure 3:**
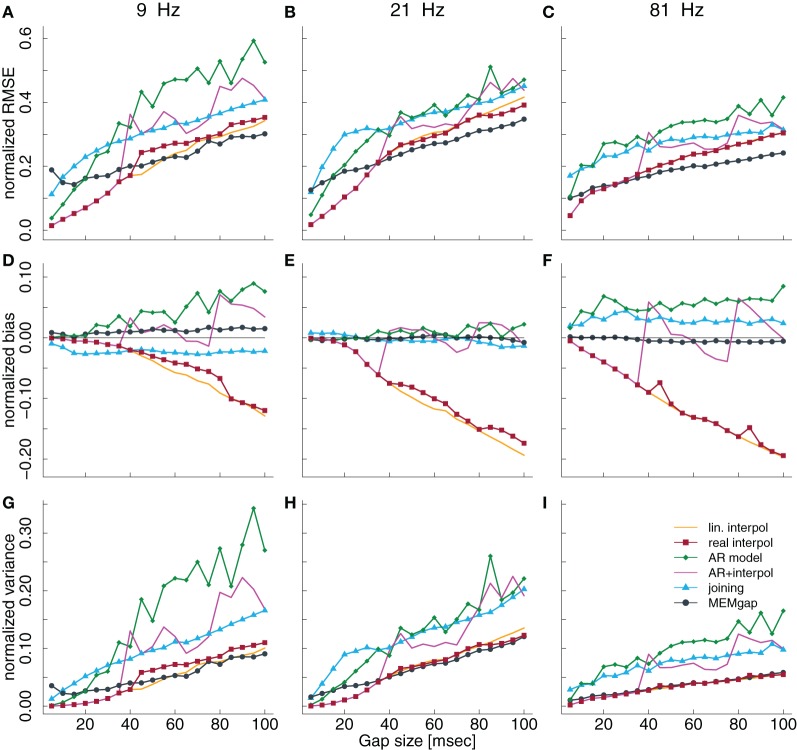
**Normalized RMSE, bias, and variance of the spectral power estimation for the frequency bin at 9 Hz **(A,D,G)**, 21 Hz **(B,E,H)**, and 81 Hz **(C,F,I)** for a model order of 32**. The colored lines illustrate the course of the normalized RMSE in **(A–C)**, the normalized estimation bias in **(D–F)**, and the normalized variance in **(G–I)** relative to the gap size for the different algorithms. The thin black line in **(D–F)** denotes an ideal estimation bias of 0.

Figures 3A–C show the influence of gap sizes between 5 and 100 ms on the error in spectral estimation for three particular frequencies (9, 21, and 81 Hz) and a model order of 32. We find that the RMSE increases with the gap size for all methods. This happens, because the information of the samples that are excluded by the gap is missing for the AR estimation, leading to a greater deviation from the AR coefficients without gaps for increasing gap size. The linear interpolation methods exhibit a negative bias and the AR-prediction shows positive bias (Figures 3D–F and 5). The negative bias of the linear interpolation methods occurs because a section of the data window is reduced to a straight line which has a power of almost 0 for higher frequencies, leading to a decrease in the estimated power for these frequencies. This effect increases with greater gaps. AR modeling can lead to jumps in the data, because the extrapolated signal from the start of the gap is not necessarily connected to the actual recorded signal at the end of the gap. Such jumps result in higher estimated power across all frequencies and thus a positive bias. For longer gaps this bias increases because the potential deviation from the true values after the gap (the jumps) becomes larger. The mixture of linear interpolation and AR prediction is in general closer to 0 than the other two, but the sign of its bias depends on data packet size and gap size. The joining and MEMgap algorithms exhibit a bias close to zero, but the RMSE is smaller for MEMgap than for joining. The variance (Figures 3G–I) also scales with gap size but there are strong differences between the methods visible, with MEMgap and the linear interpolation methods having the lowest variance.

### 3.2. Model order

**Figure 4:**
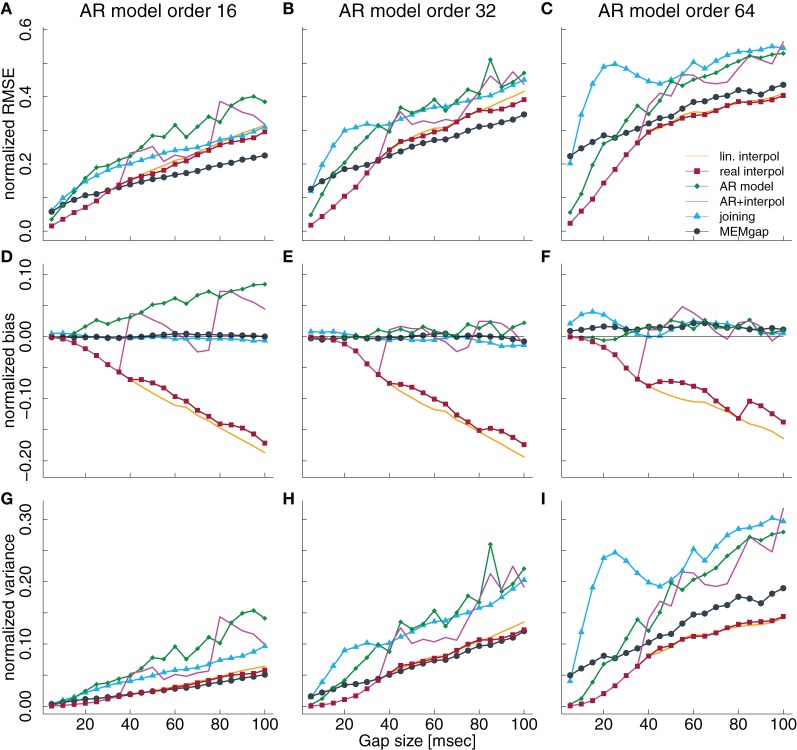
**Normalized RMSE, bias, and variance of the spectral power estimation for a model order of 16 **(A,D,G)**, 32 **(B,E,H)**, and 64 **(C,F,I)** for the frequency bin at 21 Hz**. The colored lines illustrate the course of the normalized RMSE in **(A–C)**, the normalized estimation bias in **(D–F)**, and the normalized variance in **(G–I)** relative to the gap size for the different algorithms. The thin black line in **(D–F)** denotes an ideal estimation bias of 0.

**Figure 5:**
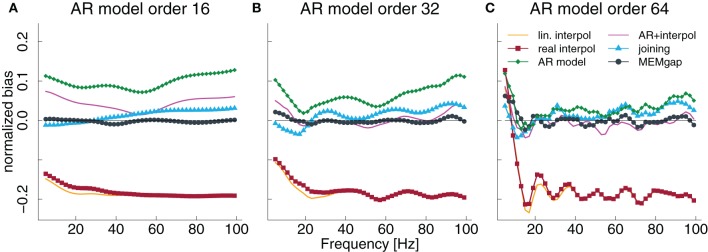
**Normalized bias for a gap size of 100 ms and an AR model order of 16 **(A)**, 32 **(B)**, and 64 **(C)** as a fraction of the power of the original signal**. The thin solid line always indicates a bias of 0.

Variations of the model order have the largest effect on the AR modeling and the MEMgap algorithm. While AR modeling exhibits a significant positive bias at 21 Hz for gaps longer than 60 ms at a model order of 16 (Figure 4D), it is not significantly biased for a model order of 32 and 64 (Figures 4E,F). As shown in section 3.3 and Figure 5, this is due to the frequency-dependency of the bias for AR modeling which has a global minimum around 20 Hz for model order 32 and 64. For MEMgap we find no significant bias for all model orders (Figures 4D–F) and that the absolute error of the power estimation, captured by the normalized RMSE, as well as the variance, increases rapidly with increasing model orders (Figures 4A–C,G–I). This is probably due to the lower number of samples fully available for AR estimation with MEMgap compared to the standard Burg algorithm: for MEMgap, forward or backward prediction errors can not be calculated for up to 2*p* samples around each gap, where *p* is the model order. Higher values of *p* only increase this difference, leaving MEMgap with less and less samples for AR estimation, thus probably leading to greater errors. In general, MEMgap has the lowest RMSE for orders 16 and 32 and gaps longer than 30 ms and the lowest RMSE of all methods with a bias close to 0 at an order of 64.

### 3.3. Frequency

In Figure 3, we show the results for low and high frequencies with 9 and 81 Hz as parts of the μ and high γ bands, respectively, in addition to the “intermediate” frequency of 21 Hz as part of the β-band. For 81 Hz, the linear interpolation methods already show a significant negative bias for gaps of 5 ms, whereas for 9 Hz this only becomes significant for gaps greater than 35 ms. This is easily understandable considering that one cycle of a 9 Hz oscillation lasts for more than 100 ms, therefore linear interpolation over a gap of 10–20 ms would be fairly consistent with the real shape of the undisturbed signal. The bias of MEMgap is not significant for any frequency (Figures 3D–F). The joining method on the other hand exhibits a negative bias for 9 Hz and gaps smaller than 40 ms and a significant positive bias for 81 Hz. For 21 Hz, The bias is significant only for gaps smaller than 10 ms. In terms of RMSE and variance (Figures 3A–C,G–I), MEMgap always displays the lowest values for gap sizes greater than 50 ms.

The results in Figures 3D–F, especially for AR modeling and joining, suggest that the bias might be frequency-dependent. In Figure 5, the bias is shown relative to the frequency bin for model orders of 16, 32, and 64 for a gap size of 100 ms where it should be most pronounced. We find that for the joining method, the bias is negative, although non-significant, for frequencies lower than 25 Hz and positive otherwise (significant for most frequencies >60 Hz). For AR modeling, the bias is in general positive (significantly for all frequencies for model order 16 and above 55 Hz for 32) and increases with frequency, has a minimum around 20 Hz for a model order of 32 and 64 and is also increased for lower frequencies. For linear interpolation, there is a bias close to −0.2, indicating a reduction in power of about 20%, for frequencies higher than 20 Hz. This value can be explained with the fact that 20% (100 ms of a 500 ms window) of the data had to be filled by linear interpolation which removes the high-frequency content. MEMgap exhibits no significant bias across all frequencies and model orders, except for very low frequencies and high model orders where all methods show a positive bias. Although the bias for the mixture of AR modeling and interpolation is also not significant for most frequencies above 10 Hz and higher model orders, this is due to the interaction between gap size and packet size for this particular method. As seen in Figures 3D or F, for example, a gap size of 80 would lead to a positive bias.

### 3.4. Application on data with stimulation

In our experiments, we received the data in packets with a length of 40 ms. This leads to the jumps seen in the bias relative to the gap size for the combination of AR modeling and linear interpolation (e.g., Figure 3F, magenta line) as either linear interpolation of AR modeling dominate the outcome. The packet length might be different for other recordings, so we excluded this method from the rest of the experiments, as the conclusions would be very specific for our setup. Further experiments are needed to investigate the influence of this specific parameter. As the simulation results of the two versions of linear interpolation did not differ much, we restricted ourselves to four of the six methods for the remainder of the paper: online-compatible linear interpolation, AR modeling, joining, and MEMgap.

A model order of 16 was chosen for spectral estimation and AR extrapolation as this section is mostly illustrative in nature and serves the purpose to study whether the results from the simulations are transferable to data with actual stimulation. In terms of the estimation bias, we found the clearest effects for a model order of 16: a negative bias for linear interpolation and a positive bias for AR modeling. The latter bias was not present for higher model orders around the studied frequency bin of 21 Hz.

**Figure 6:**
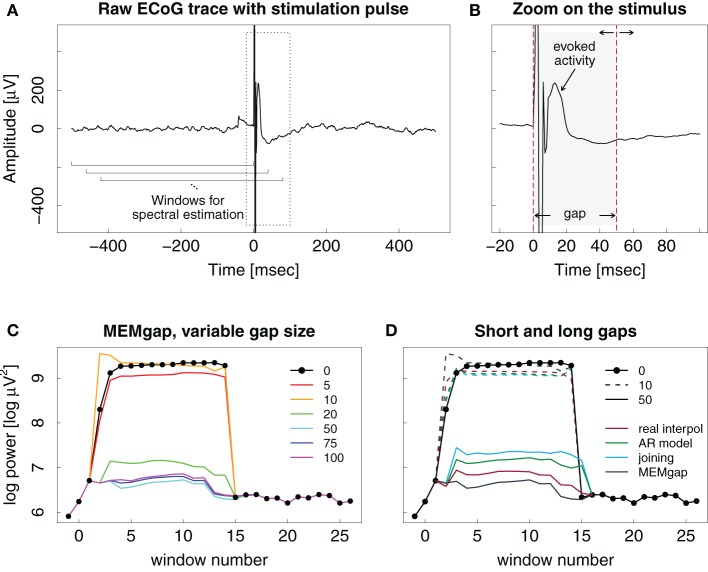
**Example ECoG trace of an epidural stimulation event**. **(A)** One second of data with the stimulation at time point 0. The brackets show the moving window used for spectral analysis. **(B)** Zoomed version of the left plot, showing the evoked activity and the stimulation artefact in greater detail. Dashed lines show the start and end markings of the gap, here with a length of 50 ms. The end point of the gap can be varied in time. **(C)** Output of the spectral estimation using MEMgap for gap sizes of 5, 10, 20, 50, 75, and 100 ms and the frequency bin centered at 21 Hz. The logarithm of the estimated power is shown because of the large differences between the power at a gap size of 0 and 50 and above. Window numbers correspond to the brackets shown in **(A)**, where the first one is 1, the second one (shifted by 40 ms) is 2 and so on. The computation of windows −1, 0, and 15–26 used data that is outside the margins of **(A)**. **(D)** Comparison of linear interpolation (red), AR modeling (green), joining (blue), and MEMgap (gray) with gap sizes of 10 (dashed) and 50 (solid) applied on the data in **(A)**. The solid black line with circles in **(C)** and **(D)** shows the result of spectral estimation without processing of the stimulation after-effect (gap size = 0).

Figure 6 illustrates the effect of epidural stimulation on the recorded ECoG activity, the evoked activity after stimulation and their influence on spectral estimation for one representative stimulation pulse. Figure 6A shows the raw trace of data with a single pulse of electrical epidural stimulation occurring at time point 0. Figure 6B displays a zoom on the first 100 ms after the pulse. The stimulation artefact itself is contained within the first 10 ms after the pulse. After that, one can find evoked activity with its peak occurring 13 ms after the pulse and an amplitude of 240 μV. This is much higher than the short-term amplitude fluctuations found in our ECoG data without stimulation.

Figure 6C demonstrates the importance of adjusting the length of the gap to the actual stimulation effects on the signal. Applying a gap of 10 ms to the data might be enough to cover the stimulation artefact itself, but the spectrum then still shows a clear positive bias due to the influence of the evoked activity. The results for short gap sizes are very similar to those without any gap. Only gaps greater than 20 ms cover the extent of the artefact and the initial evoked activity, leading to power values that are similar to those obtained for data windows without the stimulation event (windows 16–26). There is no clear difference in the outcome of the gaps greater than 20 ms.

In Figure 6D, linear interpolation, joining the data segments, MEMgap, and AR modeling are compared when applied to the stimulation event both for short and long gaps. All methods perform poorly for a gap size of 10 ms, but there are differences for 50 ms. Applying joining and AR modeling results in higher power values than linear interpolation and MEMgap with a clear difference in estimated power between the windows with and without stimulation. Assuming perfect exclusion of all stimulus-related effects, we expect that the power does not differ strongly between e.g., window 15 (which includes a small portion of the gap) and window 16 (without the gap), therefore the result of MEMgap and linear interpolation is more realistic than the output of the other methods. At least for the AR modeling method, the increased estimate of the power compared to, for example, linear interpolation is consistent with the positive bias shown in Figure 3B. A reason for the positive bias of the joining method for this example data set might be that drifts of the signal after a stimulation on epidural electrodes are common. If we take a data segment with post-stimulus drifts, exclude the gap and join the data before and after the gap into one window, it will contain a sharp discontinuity and have a comparatively high spectral power. With linear interpolation, the discontinuity will be less severe and have a smaller impact on the signal power. For MEMgap it does not play a role as data before and after the gap is always separated during estimation of the AR coefficients.

**Figure 7:**
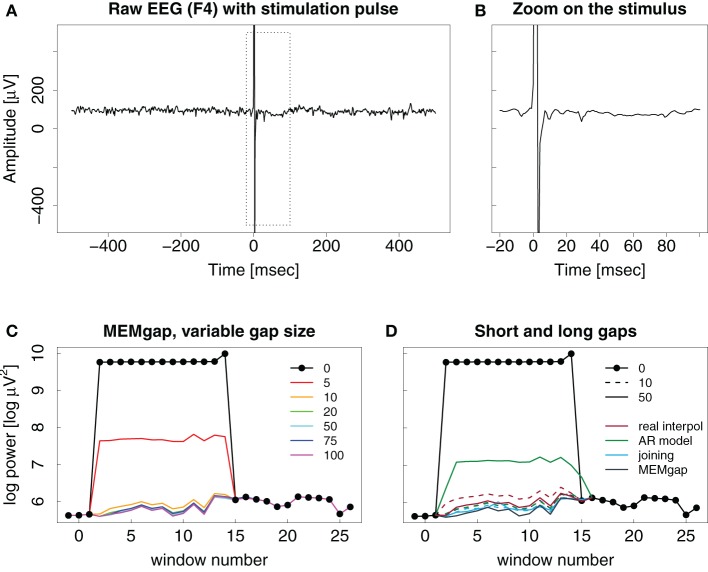
**Example trace of a TMS pulse applied over EEG channel (C4) but recorded on a distant channel (F4)**. **(A–D)** Same as in Figure 6. Note the missing evoked activity in **(A)** and **(B)** after the pulse.

Stimulation artefacts and evoked activity are found for combined EEG and TMS in a similar way as for stimulation over implanted electrodes with the strength of the evoked activity depending on the distance to the stimulation site. We illustrate this in Figure 7 with the result of a TMS pulse on the activity recorded on a distant EEG channel. There is no strong evoked potential visible after the stimulation, therefore, as is evident in Figures 7C,D, a short window of 10 ms is already sufficient to cover the artefact and to produce an estimation of spectral power that is similar in value compared to that resulting for data windows long after the stimulation when using either linear interpolation, joining or MEMgap to correct for the gap.

### 3.5. Influence on decoding performance

The stimulation-processing algorithm can bias the estimated spectrum, or will at least produce deviations from the original spectral power without gaps. This poses the question, how strongly these errors influence the actual brain-state decoding during a BCI experiment. For example, if the bias of linear interpolation toward underestimation of the signal power directly influences, how well we can differentiate data packets obtained during a movement from those recorded during rest, then this algorithm is not suitable for BSDS because it might induce a bias in the subject's performance in an online experiment.

To investigate this, we used data sets with different stimulation paradigms and recording methods (EEG and ECoG) to assess the influence of the algorithms and gap size on the decoding abilities of a BCI system. The patients always performed the same cued attempted hand movements but we varied the stimulation paradigm between no stimulation, stimulation with fixed ISI and stimulation coupled to the output of the BCI (i.e., BSDS). In the last paradigm, the stimulation pulses were only applied while the BCI detected an intention to move from modulations of the power in the β-band and therefore moved the orthosis. If stimulation was used, stimulation artefacts were identified online with a peak detector if the voltage of two consecutive samples differed by more than 1 mV. The start of the gap was set 2 ms before this artefact and the gap size was adjusted for each patient and session depending on the length of the evoked activity as determined by several test stimuli applied before the start of the session. This resulted typically in a gap length between 30 and 70 ms. Stimulus processing was performed during recording with the online-compatible linear interpolation method.

In the offline analysis, we applied the four methods: joining, linear interpolation, AR modeling, and MEMgap on these data sets and varied the gap size between 0, 10, 50, and 100. We simulated the two different stimulation conditions on the data without stimulation by varying, in which phases of the trial gaps are placed: in the uncoupled condition the whole trial was valid, so the placement of gaps was independent of the activity and brain-state of the patient. For the coupled condition only time points within the movement phase were used as gaps, thus simulating a BSDS paradigm. In both cases the ISI was fixed at 2 s.

After applying the respective stimulation processing algorithm, we computed the spectral power between 16 and 22 Hz on channels located over the right motor cortex. For EEG measurements we used FC4, C4, and CP4 as defined by the 10–10 system (Society, 2006), whereas for ECoG measurements the electrodes were selected individually per patient based on the results of a screening session. We used a window size of 500 ms and a model order of 16 for spectral estimation. These were the same parameters, channels and frequencies that had been used during the online feedback experiments in which the data was recorded. Furthermore, our simulations showed a positive estimation bias for AR modeling at 16–22 Hz only for a model order of 16, not for 32 or 64. Thus, we only used an order of 16 for the simulations on data without stimulation. In order to investigate, whether higher model orders have a substantial effect on the processing of real stimuli, we used model orders of 16, 32, and 64 on the data with open-loop and closed-loop stimulation. For each run (consisting of 11 trials), we calculated the area under the ROC curve (AUC) for the sum of the logarithm of the power values within each data window in the movement phase versus those in the rest phase. We used this as a measure of the separability of these phases on a single-packet level. Taken together from all three patients, we analyzed 87 runs of EEG recordings without stimulation, 24 runs with uncoupled EEG-TMS, 131 ECoG runs without stimuli, 51 runs of ECoG with uncoupled, and 82 runs of ECoG with coupled stimulation. For each recording and stimulation condition, algorithm and gap size, this resulted in a distribution of AUC scores, one per run.

The conditions without stimulation allowed us to test for the bias and absolute error introduced by the gaps into the AUC scores. Thus, we computed the pair-wise differences between the AUC scores of a gap size of 0 and those of all combinations of algorithms and gap size for these conditions. Using Kruskal–Wallis tests, Bonferroni-corrected for multiple comparisons, we tested which algorithm leads to the smallest absolute differences in AUC scores. We also applied Wilcoxon signed rank tests to assess, whether the median of the differences deviates significantly from 0, indicating a systematic bias in the AUC scores. As there is no “true” reference distribution of the AUC scores possible for data with stimulation, we used Bonferroni-corrected non-parametric Friedman tests which account for possible effects of using the same sessions in all conditions to test whether gap sizes greater than 0 lead to different AUC scores compared to a gap size of 0 and to test whether there is a difference between the algorithms at a certain gap size.

**Figure 8:**
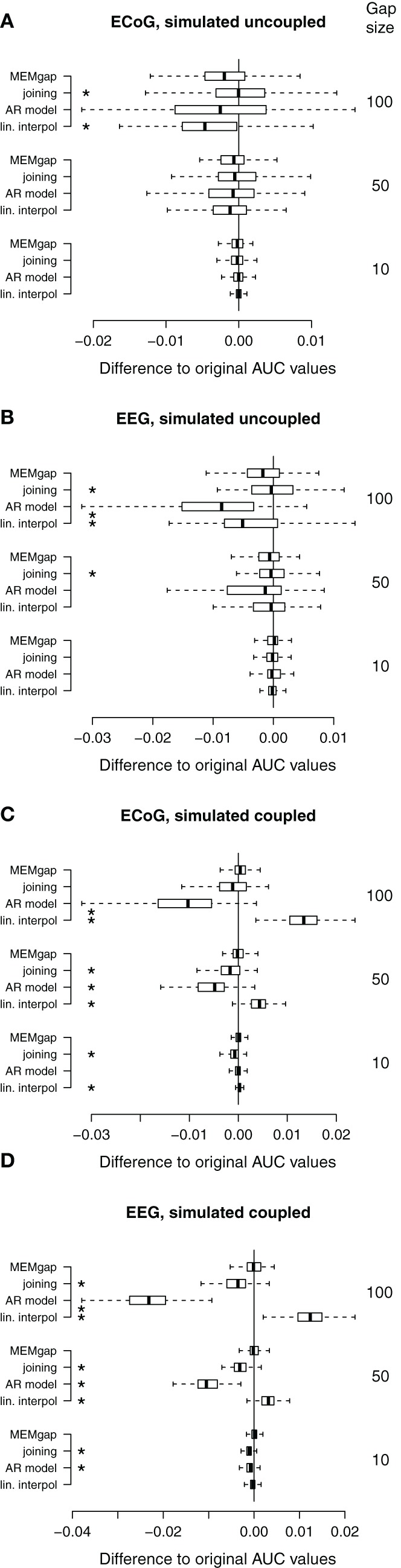
**Distributions of the differences between AUC values without gaps and AUC values of gap sizes of 10, 50, and 100 for data sets without stimulation**. A deviation from 0 indicates an over- or under-estimation of class separability. **(A)** ECoG and **(B)** EEG data with gaps simulated throughout the whole trial (*uncoupled* condition). **(C,D)** AUC values computed on the same data sets as in **(A)** and **(B)**, respectively, but with gaps simulated only within the movement phase (*coupled* condition, BSDS). Boxes cover the range between the lower and upper quartile of AUC differences with the median depicted as a black line. The whiskers extend to the most extreme data point which is no more than 1.5 times the interquartile range away from the box. ^*^AUC scores differ significantly from MEMgap for this gap size (*p* < 0.05, Friedman test, Bonferroni-corrected).

Figure 8 shows data without any actual stimulation, so ideally the difference in AUC scores between a gap size of 0 and of 100 should be zero for all runs. In Figures 8A,B, stimuli were simulated throughout the trial, thus independent of the task or the output of the BCI. Session-wise comparison of the AUC values with Friedman tests for each gap size show significant differences between MEMgap and the other algorithms only for long gaps. There is a slight decrease in the average AUC value for all algorithms for a gap size of 100 compared to 0 and MEMgap and joining yield significantly smaller absolute differences in AUC scores compared to AR modeling and interpolation (*p* < 0.000001). In Figures 8C,D, the stimuli were simulated only throughout the movement phase. We find a significant decrease in AUC values for AR modeling and a significant increase for linear interpolation. This means that linear interpolation artificially “improves” the decoding power. As shown in the simulation studies, linear interpolation of large gaps leads to a decrease of the power between 16 and 22 Hz (negative bias) which increases the event-related desynchronization effect of sensorimotor rhythms during attempted movements (Wolpaw et al., 2002). The MEMgap method shows a significantly smaller deviation of the AUC values at gap sizes of 50 and 100 from the AUC values without gaps than all the other methods (*p* < 0.000001). In contrast to the other algorithms, the median of the AUC differences after MEMgap never differs significantly from 0 for the BSDS condition, except for the ECoG data set with a gap size of 100 (*p* = 0.005). The other algorithms differ significantly from MEMgap in almost all cases of the coupled conditions.

**Figure 9:**
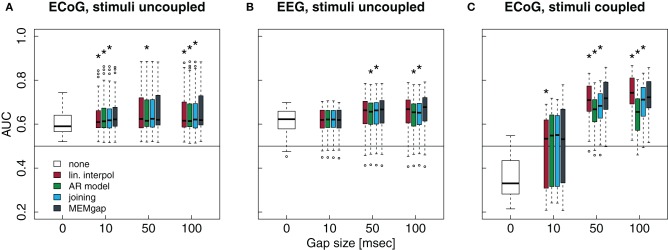
**Influence of the stimulation processing algorithm and gap size on the separability between intended movement and rest for experiments with stimulation.** No processing of the stimulation after-effects was conducted for a gap size of 0. For gap sizes of 10, 50, and 100, AUC values were calculated after application of the four algorithms (boxes from left to right) *real interpol*, *AR model*, *joining*, and *MEMgap*. A baseline AUC value of 0.5 is shown as a solid line, because this is the chance level for a purely random classifier (Fawcett, 2006). **(A)** Average AUC values for the separation of movement and rest from experiments with epidural stimulation and ECoG recordings. Stimulation pulses were given throughout the whole trial with a fixed ISI of 2 s and the gap size was varied between 0, 10, 50, and 100. **(B)** Same as **(A)**, but for TMS-EEG data with an ISI of 3 s. **(C)** Average AUC values for ECoG data sets where stimulation pulses were triggered only if the BCI system detected an intention to move within the movement phase (BSDS). Boxes are defined as in Figure 8, open circles depict AUC values outside the range of the boxplot whiskers. ^*^AUC scores for this algorithm and gap size differ significantly from MEMgap (*p* < 0.05, Friedman test, Bonferroni-corrected).

Patients in the data sets shown in Figures 9A,B were stimulated independent of the task. We show only a model order of 16, because the results for an order of 32 and 64 are very similar. It is evident from gap sizes of 0 and 10 that untreated stimulation after-effects are detrimental for decoding. Online decoding will be more successful if enough samples are excluded after a stimulus (in these examples: a gap size of 50 ms seems to work well, although this varies between patients). Using Friedman tests for session-wise comparison of the AUC scores, significant differences of the algorithms are found, although the mean absolute differences are very small (≤0.01). In case of the uncoupled ECoG condition, AUC scores with untreated stimulation after-effects are significantly lower than AUC scores for gap sizes of 50 and 100, independent of the applied algorithm (*p* < 0.01). This effect is due to residual stimulation after-effects for small or absent gaps that lead to a very high power of data windows that contain electrical or magnetic pulses. In particular such data windows in the movement phase will be classified incorrectly. If the strong after-effects are removed by longer gaps, the classifier is more likely to produce a correct result which is reflected in the increased AUC score for gaps of at least 50 ms.

Finally, in Figure 9C, stimulation was given only during the movement phase. The average AUC value for a gap size of 0 is smaller than 0.5, indicating a higher power during movement than during rest, as opposed to the expected event-related desynchronization. This is due to the task-dependent existence of the stimulation effects: the large stimulation after-effects that occur only during the movement phase lead to a very high spectral power of this phase. Thus, the spectral power of the movement phase is very well separable from the power of the rest phase for a gap size of 0. For a gap size of 10, there is a large variability in the AUC scores. This is because for one of the three patients, a gap of 10 ms was not sufficient to cover all artefact-related jumps in the recording, resulting in AUC scores lower than 0.5. If the after-effects are dealt with by using a gap size of 50, the relationship between the power during rest and feedback reverses and resembles the expected ERD/ERS pattern. For a gap size of 100, we find in Figure 9C that the largest average AUC value is reached for linear interpolation and the smallest one for AR modeling, both differing significantly from the AUC values for MEMgap (*p* < 0.000001). This relationship is found for all tested model orders, where joining and AR modeling are on average worse than MEMgap by more than 0.02 and 0.05, respectively, while linear interpolation yields higher scores by at least 0.01. This is consistent with the simulation results in Figures 8C,D indicating an artificial over- and under-estimation of class separability by these methods. It supports the hypothesis that MEMgap is probably best suited to deal with large gaps in the data, especially for BSDS, because based on the simulation studies the deviation from the true AUC value is significantly smaller than for the other methods.

## 4. Discussion

One challenge when trying to combine online brain-state decoding from spectral data and direct cortical stimulation is that the after-effects of stimulation such as artefacts (Taylor et al., 2008) or evoked activity (Matsumoto et al., 2007) can have a much higher amplitude than the background brain signals. Therefore, estimation of the brain-state from a segment of data has been unreliable, if such stimulation after-effects are contained in this segment. This leaves us with three options: we can (1) use only data segments for decoding that are free of any after-effects, (2) attempt to separate stimulation after-effects from background brain activity, e.g., by fitting a template of the expected shape of the effects to the recording, or (3) isolate the portions of the data segment that are “contaminated” by stimulation effects and use only the “clean” parts for decoding.

In earlier studies combining TMS and EEG without BSDS (i.e., without the necessity to perform real-time brain-state-decoding from the EEG), options (1) and (2) have been used. In such studies, either a fixed length window around the stimulus was removed offline (Fuggetta et al., 2006), a decomposition into artefact-free and contaminated data was attempted in post-processing (Litvak et al., 2007; Morbidi et al., 2007; Erez et al., 2010) or a sample-and-hold circuit was used during recording to fix the amplifier output at a constant level during the pulse (Ilmoniemi et al., 1997). The latter method is especially helpful for amplifiers that recover from TMS pulses only after a delay of several hundred milliseconds (Ilmoniemi and Kičić, 2010), although some current amplifiers are able to keep this delay lower than 10 ms (Veniero et al., 2009). The drawback of the sample-and-hold approach is that information on the brain signal directly after the pulse is invariably lost and that the signal contains gaps.

Option (1) has also been used by Bergmann et al. (2012) in their study on EEG-guided TMS, making a waiting period of several seconds between stimulation pulses necessary. If the brain-state is decoded from spectral features and for example 500 ms of data is needed to estimate these features robustly, one has to wait for 500 ms plus the expected duration of the stimulation after-effects for making the first estimate of the brain-state after a stimulation pulse. This duration is therefore also the absolute minimum ISI in this scenario. Removal of the after-effects by template subtraction is only possible, if several constraints are met: the full amplitude range of the stimulation effects has to be within the dynamic range of the amplifier, as portions of the data in which the amplifier is in saturation can not be recovered with this method, resulting in the necessity to correct for gaps in the signal as in option (3). If the recorded effects are not sufficiently stable, attempting to remove them will lead to residuals in the signal. Like the original after-effects, these residuals can have a detrimental effect on the quality of the estimated spectrum and, thus, the decoding process. The employed removal algorithms need to be suitable for an online BCI, so they need to work on a single-trial level and therefore should not be too computationally demanding.

We have chosen approach (3) for this work, the deliberate introduction of gaps into the signal covering the strongest after-effects of stimulation and correcting for these gaps during spectral estimation. This allows continuous decoding without influence of the stimulation after-effects, as long as the duration of the after-effects is estimated properly. To apply this approach, methods are needed that do not depend on continuous data segments for brain-state decoding and can deal with gaps in the data.

In our experiments, we analyzed the spectral power in the μ- and/or β-band to detect the patient's intention to move the paralyzed hand. We compared different approaches (linear interpolation, AR modeling, joining of data segments, and the Burg algorithm adapted for segmented data) on their ability to estimate the spectrum with gaps in the data. To this end, we used an ECoG BCI training data set and analyzed the normalized RMSE, bias and variance of the difference between the estimated spectrum with and without gaps. The RMSE increased with the gap size, although the slope of the error increase was smaller for MEMgap and joining than for algorithms that fill the gap with artificial data (linear interpolation and AR modeling). We found a clear systematic negative bias for linear interpolation and a systematic positive bias for AR modeling. We studied the frequency range between 16 and 22 Hz in most detail, where the bias of AR modeling was only apparent for a model order of 16, but a clear bias of AR modeling can be found for other frequencies at higher model orders, making this method also potentially unreliable. The joining method produces a bias close to 0 around a frequency of 20 Hz, but can lead to a positive bias for higher frequencies, whereas the MEMgap method always results in a bias close to 0. For gaps smaller than 40 ms, linear interpolation typically has the smallest absolute deviation from the true power values while MEMgap outperforms the other methods for longer gaps.

As our simulations show, the RMSE for linear interpolation is smaller than for MEMgap, thus at first glance making linear interpolation superior to MEMgap for large model orders and/or small gaps. However, in the context of a continuous BCI decoding for BSDS, the negative bias exhibited by the linear interpolation methods will bias the output of the BCI in favor of ERD, thus distorting the real performance of the participant to some extent if stimulation is coupled to the detected brain-state. We therefore think that MEMgap is most suited for BSDS as it is superior or at least equal to the other methods in terms of RMSE and variance, does not introduce a systematic bias and outperforms the other methods in minimizing the stimulation after-effects in our BCI paradigm.

Whether this approach of identifying and ignoring the segments of data dominated by stimulation after-effects is feasible in any given experimental setting depends on the duration of stimulation-evoked potentials after the pulse. As we showed here in the simulation studies, if the strongest evoked activity is contained within the first 50–100 ms after the pulse, then a decoding approach using MEMgap is feasible. If no strong evoked activity is observed, e.g., in the case of a remote recording location as illustrated in Figure 7, then a short gap of 10 ms covering the stimulus artefact together with linear interpolation or MEMgap would be sufficient. In Ferreri et al. (2011), evoked EEG activity following single pulse TMS was found for up to 300 ms after the pulse with amplitude fluctuations of less than 20 μV for late components. Although we do not expect that such small potentials would have a large impact on the estimated spectrum, especially compared to the stimulation artefact itself or early evoked activity, for every experiment of BSDS with continuous decoding, the size and shape of the evoked activity should be carefully studied to get a proper estimate of the duration of strong after-effects. As was shown by Casarotto et al. (2010), the after-effects depend on a number of parameters, such as stimulation intensity, location and (in the case of TMS) coil orientation. If gaps longer than the 100 ms tested here are necessary to cover all stimulation-related activity, one should either wait long enough until all effects have ceased before making the next brain-state decoding attempt, or increase the size of the data window on which the spectrum is estimated to ensure that it contains enough clean samples to compute a valid estimate.

In conclusion, we have shown that the application of cortical stimulation coupled to the output of an online brain-state decoder based on spectral features is feasible as long as the employed algorithms remove both the stimulation artefact and large early components of evoked activity and allow spectral estimation on non-continuous data. Especially if closed-loop BSDS is used, algorithms that do not introduce a strong bias into the estimated spectrum such as MEMgap are to be preferred over biased methods like linear interpolation to ensure a reliable decoding of the brain-state. In general, the methods investigated here are not restricted to applications with cortical stimulation but can be employed whenever spectral estimation has to be performed on non-continuous data sets with missing blocks of samples.

### Conflict of interest statement

The authors declare that the research was conducted in the absence of any commercial or financial relationships that could be construed as a potential conflict of interest.

## Figures and Tables

**Figure A1:**
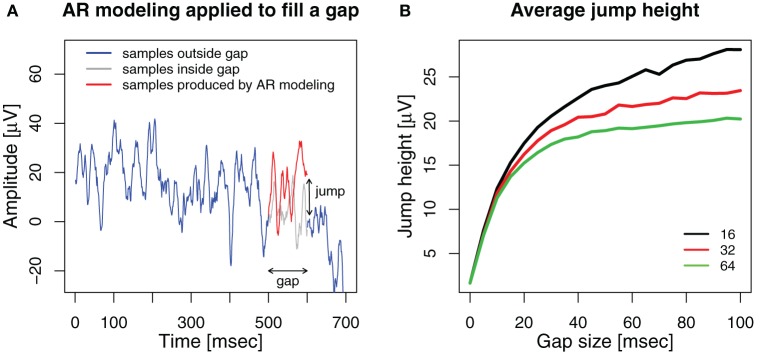
**(A)** An example, how AR modeling fills a gap in the signal. A gap is introduced into an ECoG signal (blue) between 500 and 600 ms. An AR model of order 32 is estimated from the 500 ms before the gap and applied as a linear predictor to generate 100 samples to fill the gap (red). The original ECoG samples within the gap are shown in gray. The voltage difference between the last sample generated for the gap and the first ECoG sample after the gap is the jump height. **(B)** Average jump height after filling the gap with AR modeling for model orders 16 (black), 32 (red), and 64 (green).

## 1. Appendix

### 1.1. The burg algorithm

The Burg algorithm is used for the estimation of the coefficients *c*_*i*_ of an autoregressive (AR) model

$$x(t_{p})=\sum_{i\;=\;1}^{p}c_{i}x(t_{p\;-\;i})+e$$

with order *p* for samples *x*(*t*_*k*_), 0 ≤ *k* < *N*, *e* a sample from a white noise sequence. The algorithm needs *p* recursive steps and in each step *j*, the coefficients *c*_*j*, *i*_ for an autoregressive model of order *j* are computed by the following procedure:

An initial estimation of the power of the white noise component in the AR model is obtained by

$$P_{0}=\frac{1}{N}\sum_{k\;=\;0}^{N-1}|x(t_{k}){|}^{2}.$$

Each new coefficient *c*_*i*, *i*_ is computed by minimizing the forward and backward prediction errors

$$\begin{array}{c} f_{p,k}=x(t_{k})-\sum_{i\;=\;1}^{p}c_{p,i}x(t_{k\;-\;i})\text{ with }k=p,\ldots ,N-1\\ b_{p,k}=x(t_{k\;-\;p})-\sum_{i\;=\;1}^{p}c_{p,i}x(t_{k\;-\;p\;+\;i})\text{ with }k=p,\ldots ,N-1 \end{array}$$

with the formula

(A1) $$c_{i,i}=\frac{-2\sum_{k\in I_{i}}f_{i\;-\;1,k}\cdot b_{i\;-\;1,k\;-\;2}}{\sum_{k\in I_{i}}\left(|f_{i\;-\;1,k}{|}^{2}+|b_{i\;-\;1,k\;-\;1}{|}^{2}\right)},\;I_{i}=\{i+1,\ldots ,N-1\}.$$

Each previously computed coefficient *c*_*i* − 1,*k*_ is then adjusted by

$$c_{i,k}=c_{i\;-\;1,k}+c_{i,i}\cdot c_{i\;-\;1,i\;-\;k}$$

and we update the power estimation to

$$P_{i}=(1-|c_{i,i}{|}^{2})\cdot P_{i\;-\;1}$$

and the forward and backward prediction errors:

$$\begin{array}{c} f_{i,k}=f_{i\;-\;1,k}+c_{i,i}\cdot b_{i\;-\;1,k\;-\;1}\\ b_{i,k}=b_{i\;-\;1,k\;-\;1}+c_{i,i}\cdot f_{i\;-\;1,k}. \end{array}$$

After *p* steps, this results in the AR coefficients *c*_*i*_ = *c*_*i*,*p*_, *i* = 1, …, *p*.

### 1.2. Estimating the spectrum from an AR model

An AR model can be interpreted as an all-pole infinite-impulse-response filter with order *p* and coefficients *c*_*i*_ which is applied to a white noise process with a power of *P*_*p*_ (Pardey et al., 1996). Thus, after finding the *p* autoregressive coefficients *c*_*i*_, one can estimate the spectrum by evaluating the transfer function $H(z)=\sqrt{P_{p}}{\left(1-\sum_{k=1}^{p}c_{k}z^{k}\right)}^{-1}$ of the filter to find power values

$$P(\omega )=\frac{P_{p}}{|1-\sum_{k\;=\;1}^{p}c_{k}e^{-jk\omega }{|}^{2}}$$

at (normalized) frequencies ω.

### 1.3. The MEMgap algorithm

If one assumes that a sequence *g* of length *N* exists and that *g*(*n*) = 1 only if the corresponding sample *x*(*t*_*n*_) is part of a gap in the data and 0 otherwise then we just have to make sure that none of the samples *x*(*t*_*n*_) with *g*(*n*) = 1 influence the estimation of the model coefficients. The Burg algorithm computes the AR coefficients for order *p* in *p* steps, yielding in the *i*-th step the coefficients of an AR model with order *i*. If we use in the *i*-th step only those samples fully for computation of the AR coefficients that are at least *i*+1 time steps away from a sample with *g*(*n*) = 1, we achieve the desired effect. To be more precise, the coefficients are computed by evaluating forward and backward prediction errors (see Appendix 1.1). In the MEMgap algorithm, forward prediction errors are only computed for samples that are at least *i*+1 time steps after a gap, backward prediction errors only for those at least *i*+1 time steps before a gap. Formally, this is done by modifying *I*_*i*_ in Equation (A1) to the set

$$I_{i}=\{k\;|\;g(k)=0\;\wedge \;i<k<N\;\wedge (k-n<0\;\vee \;k-n>i)\;\forall n\text{ with }g(n)=1\}.$$

This set can also be computed iteratively in each step of the Burg algorithm as *I*_*i*_ = *I*_*i* − 1_ ∩ *I*′_*i* − 1_, where *I*′_*i* − 1_ is the set *I*_*i* − 1_ with each entry incremented by 1 and *I*_0_ = {*k* | *g*(*k*) = 0 ∧ 0 < *k* < *N*}. This resembles a “forbidden zone” that initially contains only the gaps but grows in each step of the algorithm by one sample. The estimation of the white noise power *P*_0_ has to be calculated only with samples outside of gaps: $P_{0}=\frac{1}{\left|I_{0}\right|}{\sum_{k\in I_{0}}\left|x(t_{k})\right|}^{2}$. The rest of the algorithm works as the standard Burg algorithm described in Appendix 1.1.

Obviously, this method can only work if the continuous segments between gaps are long enough. Therefore, there needs to be at least one segment of samples with a length that is at least equal to the model order *p* in order to make an estimation of the spectrum with this method. In practice, it is preferable if the number of samples in such a segment is several times higher than the model order in order to reduce the bias and variance of the estimator. If there are *n*_*s*_ segments of clean data with a length greater than *p* and the total number of samples in these segments equals *M*, then only *M* − 2n_*s*_*p* forward and backward prediction errors are available in the *p*-th step of the MEMgap algorithm, although all *M* samples are evaluated to compute these errors. This means that even if the total number of samples within gaps might be the same, one can expect that the variance of the spectral estimation will be smaller if there are only a few large gaps in the data compared to having many small gaps because less samples contribute fully in the second case. According to de Waele and Broersen (2000), the same holds for the estimation bias which is inversely proportional to the number of available samples.

### 1.4. AR modeling induces jumps in the signal

The approach to fill the gap with samples that were extrapolated by an AR model fitted to the data before the gap can be problematic, if the extrapolation diverges strongly from the measured data. When actually measured samples are added to the data buffer after the gap, there can be a large amplitude difference between the last (extrapolated) sample within the gap and the first measured sample after the gap (Figure A1A). Such a “jump” in the signal results in high power across all frequencies, thus distorting the spectrum. To assess the influence of the model order and the gap size on the jumps, we ran simulations on the data from the ECoG recordings without stimulation used in Figure 8A: in total, 11266 stimuli were simulated on 397 minutes of data, the gap size was varied between 0 and 100 ms in steps of 5 ms and model orders 16, 32, and 64 were tested.

We applied AR modeling to deal with the gaps and measured the jump as the absolute voltage difference between the last sample generated by the AR model at the end of the gap and the first sample after the gap. The results are shown in Figure A1B. For comparison, the average absolute voltage difference between neighboring samples of the original ECoG data without any gaps or stimuli is 1.68 ± 0.43 μV (mean ± std).

We found that for all model orders, the average height of the jump increases sharply up to a gap size of 10, yielding 12.35 ± 5.0 μV for order 16, 11.82 ± 4.43 μV for 32 and 11.29 ± 4.37 μV for 64. For further increasing gap size, the average jump height increases more slowly for higher model orders than for lower ones. For a gap size of 100 ms we find average jump heights of 28.08 ± 14.94 μV for order 16, 23.45 ± 12.51 μV for 32 and 20.23 ± 11.94 μV for 64. Thus, while the jump height at the end of the gap is significantly smaller for a model order of 64 compared to 32 and 16 (gap size = 100, paired Wilcoxon signed rank tests, both *p* < 10^−17^), it is still vastly higher than the average sample-to-sample difference for ongoing ECoG activity. Therefore, we cannot conclude that higher model orders prevent jumps after the gap.

Furthermore, if the gaps are used to cover the effects of real stimulation, there has to be a jump at the end if the gap is filled by AR modeling and evoked activity is present. AR modeling attempts to extrapolate the pre-stimulus signal which almost certainly differs in its time course from the stimulation-evoked activity, therefore extrapolation can not work perfectly, regardless of the model order.
