# Erratum: Coupling BCI and cortical stimulation for brain-state-dependent stimulation: methods for spectral estimation in the presence of stimulation after-effects

**DOI:** 10.3389/fncir.2013.00006

**Published:** 2013-02-11

**Authors:** Armin Walter, Ander Ramos Murguialday, Martin Spüler, Georgios Naros, Maria Teresa Leão, Alireza Gharabaghi, Wolfgang Rosenstiel, Niels Birbaumer, Martin Bogdan

**Affiliations:** ^1^Department of Computer Engineering, Wilhelm-Schickard-Institute, Eberhard Karls University TübingenTübingen, Germany; ^2^Institute of Medical Psychology and Behavioural Neurobiology, University Hospital TübingenTübingen, Germany; ^3^Health Technologies Department, TECNALIASan Sebastian, Spain; ^4^Department of Neurosurgery, University Hospital TübingenTübingen, Germany; ^5^Department of Neuroprosthetics, Centre for Integrative Neuroscience, Eberhard Karls University TübingenTübingen, Germany; ^6^IRCCS, Ospedale San CamilloVenice, Italy; ^7^Department of Computer Engineering, University of LeipzigLeipzig, Germany

The article “Coupling BCI and cortical stimulation for brain-state-dependent stimulation: methods for spectral estimation in the presence of stimulation after-effects” (Walter et al., 2012) incorrectly omitted four authors. The list of authors should have been: Armin Walter, Ander Ramos Murguialday, Martin Spüler, Georgios Naros, Maria Teresa Leão, Alireza Gharabaghi, Wolfgang Rosenstiel, Niels Birbaumer, Martin Bogdan.

All further references to this paper should cite the corrected author list as displayed above.

Furthermore, the text of the “Acknowledgments” section needs to be changed to: This work was funded by the European Research Council (ERC) grant 227632 “BCCI—A Bidirectional Cortical Communication Interface” and the Reinhardt-Koselleck Award of the Deutsche Forschungsgemeinschaft (DFG). We thank Alexandros Viziotis for his work with the patients and the two reviewers for helpful comments on the manuscript.
